# Visualization of the distribution of covalently cross-linked hydrogels in CLARITY brain-polymer hybrids for different monomer concentrations

**DOI:** 10.1038/s41598-022-17687-x

**Published:** 2022-08-08

**Authors:** Andrey V. Malkovskiy, Ariane Tom, Lydia-Marie Joubert, Zhenan Bao

**Affiliations:** 1 Carnegie Institute for Science, Stanford, CA 94305 USA; 2 Department of Bioengineering, Stanford University, Stanford, CA 94305 USA; 3 Cell Sciences Imaging Facility (CSIF), Stanford University, Stanford, CA 94305 USA; 4 Department of Chemical Engineering, Stanford University, Stanford, CA 94305 USA

**Keywords:** Biophysical chemistry, Neuronal physiology, Micro-optics

## Abstract

CLARITY is a tissue preservation and optical clearing technique whereby a hydrogel is formed directly within the architectural confines of ex vivo brain tissue. In this work, the extent of polymer gel formation and crosslinking within tissue was assessed using Raman spectroscopy and rheology on CLARITY samples prepared with a range of acrylamide monomer (AAm) concentrations (1%, 4%, 8%, 12% w/v). Raman spectroscopy of individual neurons within hybrids revealed the chemical presence and distribution of polyacrylamide within the mouse hippocampus. Consistent with rheological measurements, lower %AAm concentration decreased shear elastic modulus G’, providing a practical correlation with sample permeability and protein retention. Permeability of F(ab)’2 secondary fluorescent antibody changes from 9.3 to 1.4 µm^2^ s^−1^ going from 1 to 12%. Notably, protein retention increased linearly relative to standard PFA-fixed tissue from 96.6% when AAm concentration exceeded 1%, with 12% AAm samples retaining up to ~ 99.3% native protein. This suggests that though 1% AAm offers high permeability, additional %AAm may be required to enhance protein. Our quantitative results on polymer distribution, stability, protein retention, and macromolecule permeability can be used to guide the design of future CLARITY-based tissue-clearing solutions, and establish protocols for characterization of novel tissue-polymer hybrid biomaterials using chemical spectroscopy and rheology.

## Introduction

CLARITY is a two-step chemical process that alters intact neural tissue such that it becomes optically transparent and macromolecule-permeable^1^. In the first step, a hydrogel is synthesized inside of brain tissue. This hydrogel forms an internal scaffold, which acts to preserve the tissue’s native shape and bio-architecture with micron-scale precision. In the second step, lipids that are unable to bind to the hydrogel are cleared from the tissue either through electrophoretic tissue clearing (ETC) or passive thermal diffusion (passive CLARITY)^2^, leaving behind a hydrogel-tissue hybrid that retains most of the biological information while amenable to advanced imaging techniques for investigation of complex neural wiring.

Rather than simply applying a refractive index matching solution, e.g. urea or glycerol, to fixed tissues^3–5^, CLARITY was the first aqueous solution-based clearing technique to use a hydrogel that stabilized proteins so that light-dispersing components could be removed^6^. This novel approach significantly enhanced the quality of transparency and molecular phenotyping for larger tissue samples, allowing high-resolution 3D-imaging of whole mouse brains at rapid speeds^2^. Importantly, it demonstrated that whole, intact neural tissue could be seamlessly interwoven with a soft synthetic material without harming the native biological architecture. This represented a significant development in fields of neuroscience and connectomics, as well as materials/chemical engineering, providing inspiration for fully integrated polymer-based neural interfaces.

**Figure 1:**
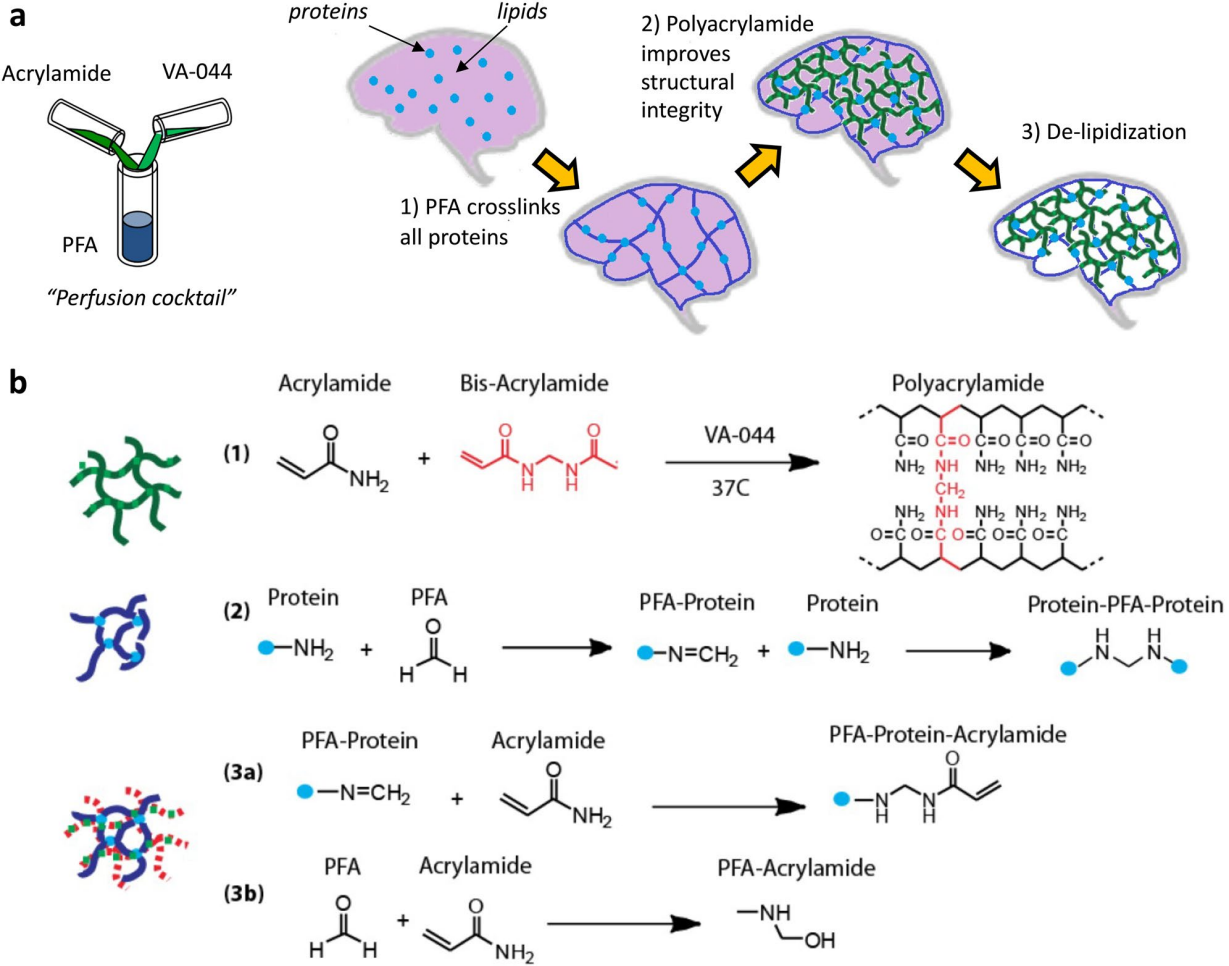
(**a**) Schematics of the CLARITY polymer tissue hybridization process: (1) in vivo transcardial perfusion of reagents into tissue containing native protein (blue circle). Tissue excised. (2) Ex vivo polymer tissue hybridization is initiated by raising temperature of degassed solution to 37 C. Thermal initiator catalyzes radical chain polymerization of monomer (green lines), and PFA (blue lines) binds to amine groups. (3) Tissue clearing by surfactant solution removes physically unbound reagent material and light scattering lipids. (**b**) Chemical reaction schemes occurring during CLARITY hybridization: (1) typical polyacrylamide hydrogel is formed via a free radical polymerization reaction amongst acrylamide and bis acrylamide monomers. (2) Typical tissue fixation using paraformaldehyde occurs when aldehyde reacts with amine groups of proteins. (3a) During CLARITY hybridization, PFA modified proteins may react with acrylamide to form PFA Protein Acrylamide linkage, to which other acrylamide monomer units can bind during addition steps. (3b) Additional reactions may occur amongst reactive precursor solution components, such as the reaction between PFA and acrylamide.

As described by Chung et al.^6^ in the first iteration of the CLARITY technique, key components of this solution are paraformaldehyde (PFA), acrylamide monomer (AAm), bis-acrylamide cross-linker, and a radical initiator 2,2′-Azobis[2-(2-imidazolin-2-yl)propane]dihydrochloride (VA-044), which are delivered to the tissue via transcardial perfusion prior to extraction (Fig. 1A). Once fully diffused into the tissue, acrylamide is triggered to polymerize by exposing the tissue to slightly elevated temperature at 37 °C. The result is a combination of PFA-fixed brain tissue and polyacrylamide ‘skeleton’: the CLARITY hybrid between brain and polymer components.

Understanding the chemical interactions between synthetic material and tissue is crucial for tailoring physical properties of the resulting composite. The ideal CLARITY hybrid should possess a minimum level of crosslinking that will retain as much of the native biological species during clearing as possible, and still offer rapid diffusion rates of small molecules for immunolabeling^7^, highlighting a tradeoff between mechanical stability and permeability.

There have been several reports that describe the augmentation of the CLARITY protocol, seeking to optimize properties^2,8–11^. Although these methods have been rigorously verified using the confocal fluorescence microscopy techniques, the material properties of the hybrids resulting from these and of CLARITY systems have nonetheless not yet been systematically quantified. The first attempt to systematically explore the optimization of tissue transparency and rigidity in CLARITY hybrids was conducted by Epp et al.^12^, who modulated the acrylamide/PFA/bis-acrylamide ratios and temperature at which ETC clearing occurred, but only light transmission and fluorescence image resolution were measured. In addition, the acrylamide/PFA/bis-acrylamide concentrations were only tested in the narrow range of from 4/4/0.05% to 3/3/0.025%. Thus, a deeper understanding of how precursor solution parameters affect hydrogel network density, tissue permeability, protein loss, and distribution of polymer within subcellular and cellular tissue has not been obtained.

In this work, we investigate the effects of the various chemically-reactive entities within the polymer-tissue composite. We present spectroscopic and rheological evidence that synthesis of a covalently bound polyacrylamide network occurs throughout brain tissue, and provide a better understanding of the how parameter variation affects physiochemical properties of CLARITY tissue-polymer hybrids material.

## Results and discussion

The CLARITY hydrogel tissue-polymer hybrid, unlike those produced by other similar brain-clearing techniques for improving brain transparency for visible light optical techniques, possesses superior protein retention and minimal disturbance of the original neural network. The stability and integrity allow it to be immunostained by perfusing antibodies for subsequent imaging. The structural integrity can be improved by altering the concentrations of the initial reaction mixture compounds. However, the major question of how such modifications of the procedure affect the microstructure and distribution of the polymer network throughout the tissue have not been answered yet. In this paper, we use correlative fluorescence and Raman spectroscopy imaging to address this problem.

We first decided to characterize the mechanical properties of CLARITY hybrids. The mechanical behaviour and permeability of hydrogels is dependent on the microstructural architecture, which is influenced by factors such as the molecular weight of the polymer, monomer concentration, and concentration of the crosslinking agent^13^. In this work, we assessed viscoelastic properties of CLARITY hybrids by varying the monomer concentration from 1 to 12wt% AAm, while maintaining the same monomer to crosslinker molar ratio at 142:1 and VA-044 thermal initiator at 0.25% concentration.

Rheology of polyacrylamide systems as a function of varying monomer and crosslinker has been well studied^14–16^. The mechanism of typical free radical polymerization can be explained by the Flory–Stockmayer theory of gelation^17^, whereby a monomer unit may bind to other monomer and crosslinker units to form longer chains (Fig. 1, reaction 1). However, during ex vivo free-radical chain polymerization of polyacrylamide in brain tissue, additional reactions may occur (Fig. 1, reactions 3a, 3b). In CLARITY hybrid systems, acrylamide can participate in free-radical polymerization, as well as form covalent bonds^18^ with the paraformaldehyde, which may be bound to the proteins (Fig. 1, reaction 2).

Multiple rheological studies of freshly dissected and unfixed brain tissue have been performed^19–22^, however, rheology of fixed brain tissue as well as other tissue biopsies is not common, because samples are often small, anisotropic, and irregularly shaped. To address these issues, we modified typical rheology protocol on a commercially available instrument to increase the measured torque to values above noise levels by using a custom-machined 3-mm parallel plate, and preparing tissue punches 2 mm thick and 6 mm in diameter, with a gap size of 0.8 mm. The compression and overflow of the sample from beyond the edges of the plate created a more uniform grip reducing slippage during experiment, and offset the sample radially, such that the sample yielded a higher torque at a given stress. Measurements were conducted while maintaining sample hydration in 1X PBS at all times to reduce edge effects of sample dehydration.

**Figure 2:**
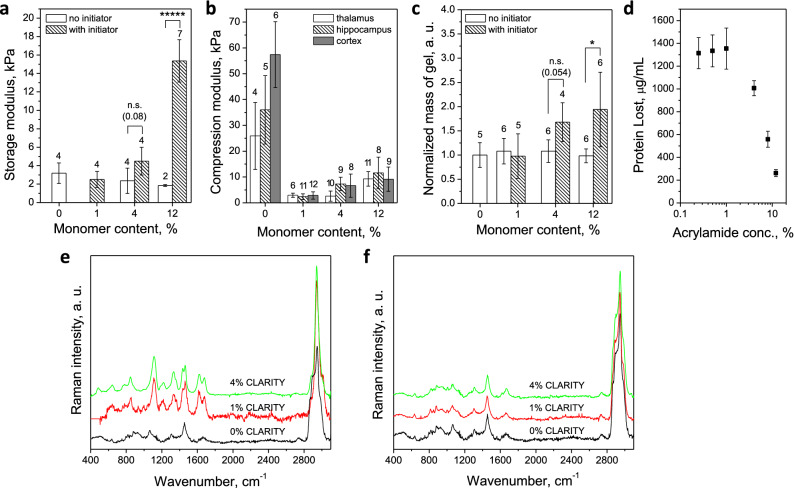
Effect of initiator and different monomer concentrations on rheology and chemistry of CLARITY hybrids. (**a**) storage modulus averaged from 0.1 to 1 Hz and 26 C vs. monomer content, with initiator (hashed bars) and without (hollow bars), (**b**) compression modulus of thalamus (hollow bars), hippocampus (hashed bars) and cortex (filled bars) with increasing monomer content, (**c**) normalized to control sample mass of gel relative to monomer content, with initiator (hashed bars) and without (hollow bars), (**d**) protein loss measured as protein concentration (μg ml^−1^) in 4% SDS surfactant solution rinsate after 14 days of washing at 37 C as a function of increasing % AAm (n = 8 across 4 animals), (**e**) Raman spectra of cleared brain tissue prepared with 0, 1 and 4% AAm and initiator, (**f**) Raman spectra of cleared brain tissue prepared with 0, 1 and 4% AAm and no initiator. For **a**, **b**, **c** number of samples marked on top of each bar. Error bars mark means SD. Statistical significance marked as follows: *(p < 0.05), *****(p < 0.00001), as measured by 2 sided non parametric Mann Whitney U test.

In order to verify whether CLARITY hybridization incorporates a polyacrylamide network with brain tissue, CLARITY hybrids were made with and without VA-044 radical initiator. Rheology data, normalized mass of gel, as well as chemical composition data (Fig. 2) show there is no gel formation when all reaction mix components are added without the initiator. Conversely, storage modulus and retaining mass of cleared brain increases when initiator is part of the reaction mix. The forming gel was sufficiently bound to tissue so that the chains were not rinsed away during clearing and the amount of lost protein falls sharply with increasing monomer concentration above 1% (Fig. 2c,d). Chemically, the C–C backbone Raman peak intensity at 1110 cm^−1^ increases with %AAm concentration, in agreement with literature^23,24^.

The viscoelastic behavior of CLARITY hybrids, namely a comparison between storage modulus, G′, and loss modulus, G″, can be used to assess the degree of crosslinking. Figure 2a shows that for all hybrids synthesized with 1%, 4%, and 12% AAm, G′ exceeds G″ at low frequencies, indicating at least average degree of crosslinking. Compression modulus (Fig. 2b) also increases with increasing %AAm across all brain parts: cortex, thalamus and hippocampus. When we performed frequency sweeps of moduli (Supplemental Fig. 1), it became apparent that the loss moduli G” are shifted slightly to higher frequencies with increasing %AAm, suggesting that the degree of crosslinking decreases, but not substantially. The gels exhibited outstanding temperature stability with minimal changes in storage moduli values in the range of 25–100 °C, unlike the PFA-only fixed brain tissue (Supplemental Fig. 2).

**Figure 3:**
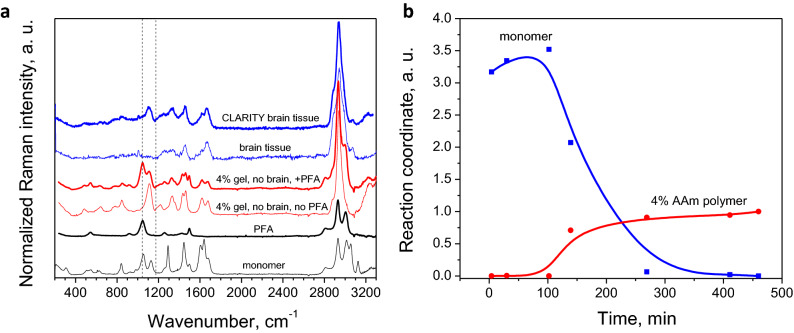
Formation of brain-polymer hybrid ex vivo, monitored by Raman spectroscopy in a wide spectral range: (**a**) Raman spectra of monomer (black line), PFA (thick black line), 4% AAm PA gel without PFA (thin red line) and with PFA (thick red line), brain tissue (thin blue line) and CLARITY brain tissue (thick blue line). Spectra vertically offset for ease of viewing. Raw data was corrected by subtracting the water signal and fluorescence background. (**b**) Relative intensities over reaction time of monomer (blue dots) and 4% AAm PA gel (red dots) peaks as fitted by Lorentzians. Solid lines are guides for the eye.

Spectroscopically, we assessed the reaction kinetics, by monitoring the transition of monomer to polymer over time in situ (Fig. 3). We were able to improve precision of quantitative spectroscopy by applying multivariate statistical methods to the full spectrum of our data. Simultaneous inclusion of multiple intensities generates a signal averaging effect, greatly improving the quality of the spectral fits^25^. This method models the experimental curve at each pixel as a linear combination of the curves of known components (in this case, tissue and polymer), in order to predict composition within unknown samples. The quality of fit with this method is demonstrated in Supplemental Fig. 3. Upon polymerization, the monomer C=C and C=O bonds are converted to C–C and C–O^24^, and the C–CH peak at 1290 cm^−1^ is minimized as end groups of acrylamide are reacted^26,27^ (Fig. 3a). For detailed peak assignments, refer to Supplemental Fig. 4. The conversion of monomer to polymer was tracked (Fig. 3b) by fitting curves at each time point to curves of pure acrylamide monomer solution (t = 0) and 4% polyacrylamide gel (t = ∞). Our results indicate that complete reactivity is achieved at 5 h at room temperature and imply that reacting the solution at elevated temperature 37 °C for at least 4 h, as suggested by the published CLARITY protocol, should reach equilibrium. The gel precursor samples are initially stable during the first minutes at 37 °C for mouse perfusion (Supplemental Fig. 5). Their viscosity does not deviate from that of water. However, after 9 min, the 12% AAm sample viscosity increases by 3 orders of magnitude. This indicates that the perfusion should be accomplished in the first 5–7 min to avoid clogging issues. This was indeed the case for all experiments reported here.

**Figure 4:**
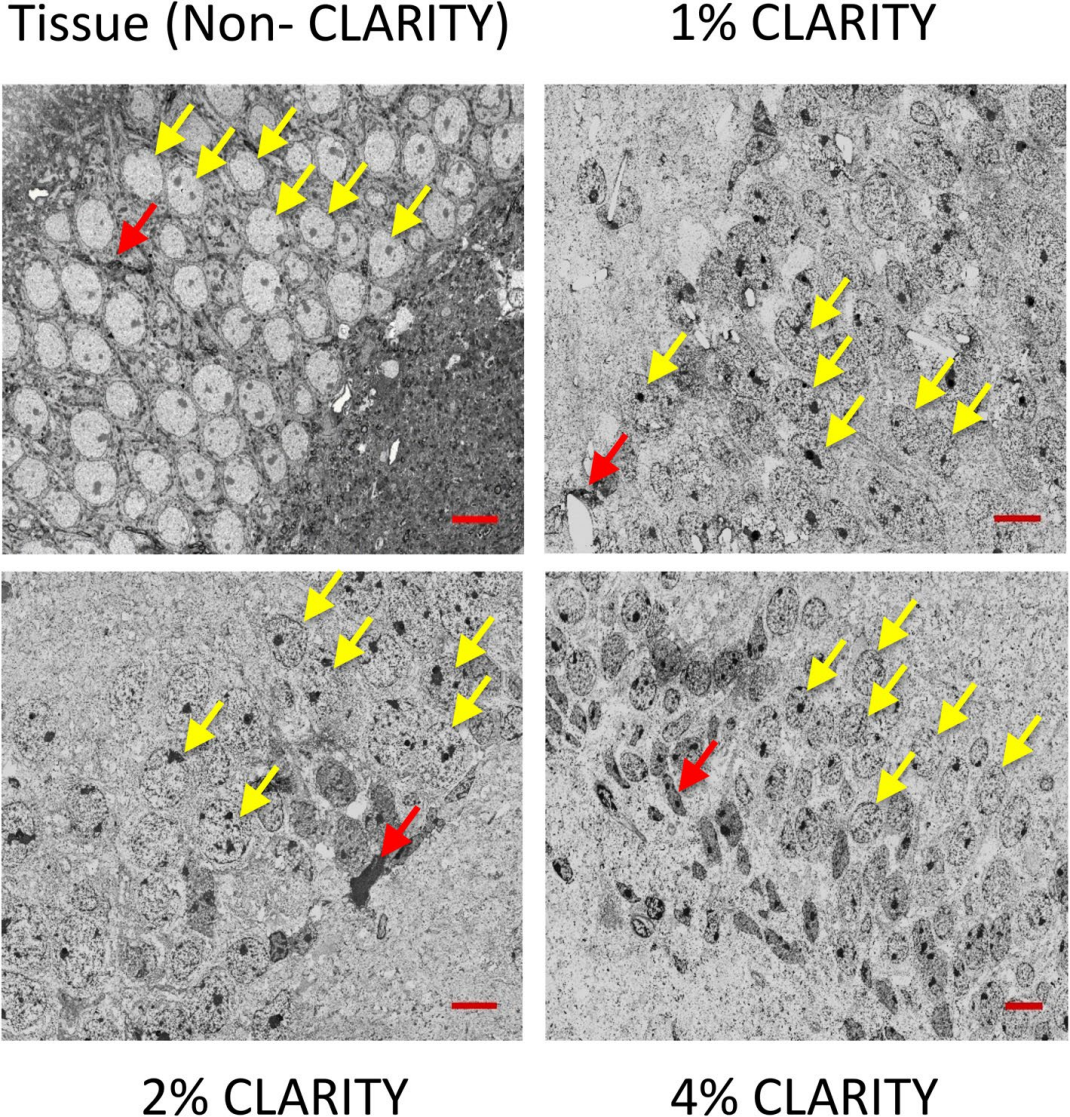
Comparison of preserved cellular morphology at different AAm concentrations (shown in subpanel labels) in SEM images of 200 nm sections of CA1 pyramidal region. Yellow arrows mark granule cells, red mark glial cells. Scale bar is 10 μm.

SEM images of 200 nm sections show preserved cellular morphology compared to non- CLARITY tissue, which looks very similar at different AAm concentrations (Fig. 4). Since the CLARIY hydrogel infiltrates the entire sample, with removal (‘clearing’) of mainly the lipid component of cells, it is predominantly the protein component of the cell that is visible, with variation in electron density, after applying different heavy metal stains. In developing a staining protocol to visualize CLARITY samples with electron microscopy, we found that initial staining with uranyl acetate enabled the additional staining with heavy metals (osmium tetroxide, lead citrate, etc.) that generally stain the lipid component of cells, presumably by acting as a 'mordant' to which other stains adhere. Darker and lighter areas therefore indicate different cell types based on the density of their cytosol, similar to electron micrographs of conventional cells. Completely unstained areas may have a lower protein component, or areas with less dense cytoplasm and vacuoles included.

**Figure 5:**
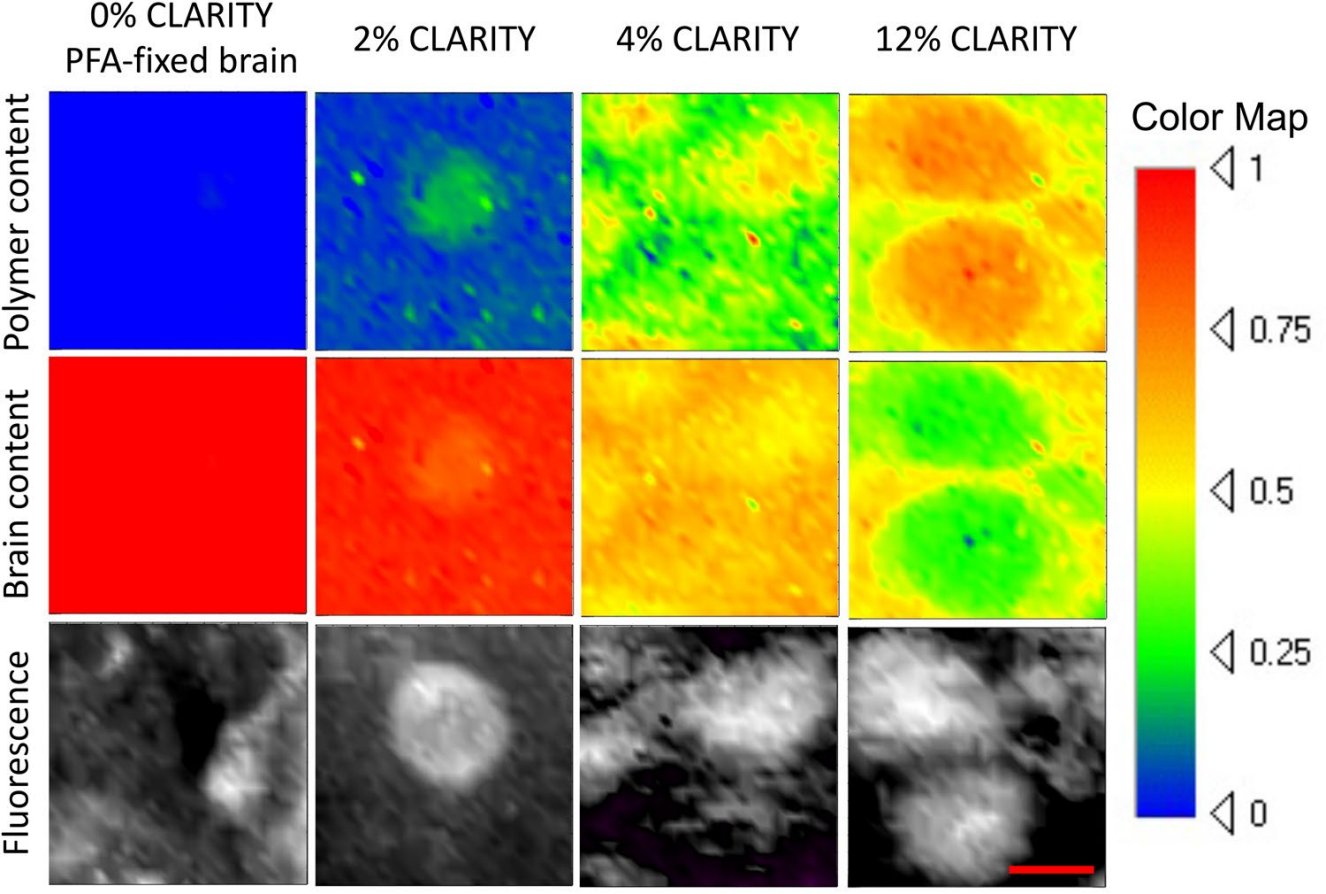
Confocal Raman maps (30 × 30 μm, 60 s per pixel, 1 μm step size, 2 mW laser power) of brain and polymer obtained using MLR analysis, correlated to confocal fluorescence images (30 × 30 μm cropped from 100 × 100 μm, 0.1 s per pixel, 1 μm step size, 2 mW laser power) of the same areas taken with the same Raman spectrometer immediately prior to Raman mapping, for non CLARITY PFA fixed and different AAm concentrations of CLARITY PFA fixed mouse brains. Brain tissue and polymer content together make 100% (i.e. 1 on color map), after correction for water signal. Confocal mapping was performed at least 10 μm away from cover glass slide using 100X 0.7 NA objective with 6 mm working distance. Scale bar 10 μm.

To map the chemical composition of the CLARITY hybrids, 250 μm tissue slices were immunostained with fluorescent contrast against parvalbumin and mounted as described in the Methods section. After fluorescence signal was collected to visualize cells, confocal Raman spectral maps of the same 30 × 30 μm^2^ area were generated by collecting the spectrum at each pixel. The spectrum of each pixel was analyzed using multiple linear regression (MLR) fitting to curves of pure polyacrylamide, protein, and water. The 2D rendering of the spectral data corresponding to the polyacrylamide content detected in parvalbumin-stained PFA-fixed tissue (no hydrogel control) and various concentration (2, 4, 12%) CLARITY samples, post-MLR fitting, are shown in Fig. 5. Maps of polymer distribution show that the polymer is present both inside and outside of Parvalbumin-stained cells of the CA1 pyramidal section of the hippocampus. Polymer content is sometimes occasionally erroneously detected in non-CLARITY fixed brain as visualized by faint green color, showing the typical level of noise to be expected in the CLARITY brain data. The 0% sample tissue was uncleared to retain structural integrity and the fitting was performed with fat signal used as an additional MLR component to improve fit quality. Interestingly, the maps of 2%, 4% and 12% samples are not homogeneous and consistently reveal stronger polymer signal at locations corresponding to cell positions in the fluorescence maps.

**Figure 6:**
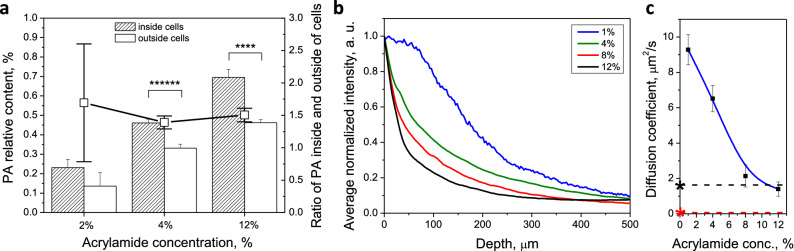
(**a**) Polymer relative content inside (hashed bars) and outside (hollow bars) cells as a function of acrylamide monomer concentration. Black line with square dots shows the ratio of polymer content inside to outside (3, 6, 5 cells analyzed for 2, 4 and 12% AAm conditions, respectively), (**b**) diffusion of F(Ab)′2 over section depth after 1 h—integrated intensity across vertical sections of confocal images, (**c**) diffusion coefficient (μm^2^ s^−1^) of F(ab)′2 fluorescent secondary antibodies as a function of increasing AAm concentration (n = 4 across 2 animals). Diffusion coefficients for un-cleared and cleared PFA-only-fixed brain tissues are marked by red and black asterisks, respectively. Dashed lines are guides for the eye. Error bars mark means ± SD. Statistical significance marked as follows: **(p < 0.01), as measured by 2-sided non-parametric Mann–Whitney U-test.

Polymer relative content was calculated by using content values from averaging 10 points inside and 10 points outside of cells (Fig. 6a). The ratio of polymer inside cell region to outside cell region remains relatively consistent for different initial AAm concentrations, and is approximately 1.5, suggesting there is 50% more polymer inside the cells than outside. This may be due to the higher concentration of proteins within the cell body, to which the polymer would bind. If polymer is indeed co-localized with proteins, this implies the interaction of polymer and formaldehyde-protein complexes hypothesized by the original CLARITY protocol (Fig. 1, reaction 3a) exists, resulting from the nucleophilic addition of the amine in proteins to the formaldehyde group.

These discrepancies in polymer distribution have additional implications on cellular tissue permeability. Permeation depth and the diffusion coefficients of 110 kDa secondary antibody F(ab)′2 fragment are plotted in Fig. 6b,c. Not only does higher amount of polymer, via higher initial monomer load, lower permeability^28,29^, but elevated polymer content within cells may result in different permeability of such areas by fluorescently-tagged molecules and may explain previous results^30,31^, which found that certain antibodies are not effective in CLARITY immunofluorescent staining, due to inability of small molecule to penetrate certain subcellular regions or reach proteins of interest. Precursor solution viscosity is very low and thus would not contribute significantly to diminished permeability. It might be useful to apply correction factors when analyzing such results from inside and outside cells. It is possible that different neuronal cells could be permeated differently, so Raman studies of PA gel in cells of different regions may be beneficial.

## Conclusions

In this study, we validated the extent of polymer crosslinking and the resulting microscopic features in CLARITY tissue-polymer hybrids using a unique combination of Raman spectroscopic and rheological techniques. Our results provide an additional reference of crosslinking within such complex structures that enables researchers to infer differences amongst sample preparation that may improve efficiency of tissue clearing techniques (e.g., reducing time it takes for samples of various sizes/morphologies to be cleared), permeability for immuno-staining or other chemical methods applied to intact tissue, and development of tissue-polymer hybrids that are more tailored to interrogate structure and function of specific biological systems where cellular or protein density is variable.

Rheology observations and protein retention experiments suggest that a minimum of 1% AAm is required to enhance protein retention above the levels of PFA-fixation only. Despite this, a lower AAm concentration increased permeability, representing a trade-off that can be tailored depending on desired purposes. Thus, our results suggest between 2 and 4% AAm to be the optimal concentration of hydrogel precursor solutions for most applications, which agrees well with conclusions from another study^32^, where 2% acrylamide monomer gels were considered best for maintaining fluorescence signal retention of immunolabelled proteins. Raman scattering confirms hydrogel synthesis and the formation of a crosslinked network ex vivo within brain tissue, which appears to be enriched within interneurons in the hippocampal region, the degree of enrichment insensitive to the monomer concentration. Thus, taken together, this study provides important insights to CLARITY hybrid properties and our described methodologies will greatly aid the quantitative assessment of other biologically-relevant polymer-embedded composites.

## Materials and methods

This study was conducted according to ARRIVE guidelines for animal-related research.

### Preparation of hydrogel precursor solutions

Hydrogel precursor solutions intended for tissue hybridization were prepared for a range of acrylamide monomer (AAm) concentrations of 1 w/v % (0.142 M), 4 w/v % (0.570 M), 8 w/v % (1.13 M), and 12 w/v % (1.71 M), according to a published CLARITY protocol (Chung et al.^1^; Tomer et al.^2^). All solutions contained 4 w/v % (1.89 M) of paraformaldehyde (PFA), 142:1 molar ratio of acrylamide to bis-acrylamide, and 0.25 w/v % (7.5 mM) VA-044 thermal initiator. For a 400 ml solution, appropriate volumes of 40 w/v % acrylamide (Bio-Rad, Hercules, CA), 2 w/v % bis-acrylamide (Bio-Rad, Hercules, CA), and de-ionized water were combined with 40 ml of 10 × PBS, 100 ml of 16 w/v % paraformaldeyhyde (Electron Microscopy Sciences, Hatfield, PA), and 1 g of VA-044 (2,2′-Azobis[2-(2-imidazolin-2-yl)propane]dihydrochloride) thermal initiator (Wako Chemicals USA, Richmond, VA). The total solution was divided into ten 40 ml aliquots in 50 ml Falcon conical tubes and stored at − 20 °C. To generate traditional polyacrylamide samples, additional batches of precursor solutions were prepared similarly, but without PFA.

### Transcardial perfusion surgeries

Adult female wildtype mice (Strain C57BL/6J #664, 9–12 weeks old, Jackson Laboratory, ME, USA) were anaesthetized with Beuthenasia-D (Merck) at 100 mg kg^−1^ body mass and perfused transcardially with 20 ml of ice-cold 1X PBS followed by either 20 ml of 4 w/v % PFA, for non-hybridized samples, or 20 ml of thawed hydrogel precursor solution, for hybridized samples, using a 26.5-gauge needle and a peristaltic pump at a speed of 0.20 ml s^−1^ (approx. 2 min). Brains were harvested and samples that would be hybridized with polymer using the CLARITY protocol were incubated in the remaining 20 ml of precursor solution at 4 °C for 3 days.

### Polymer-tissue hybridization via CLARITY

To initiate polymerization of the gel via thermal-initiated free radical polymerization, oxygen was first purged from the conical tubes and replaced with nitrogen gas using a standard vacuum pump and desiccation chamber for 10 min. Next, conical tubes containing the samples were placed on a shaker in a 37 °C warm room for 4 h. After polymerization, CLARITY whole-brain samples were gently removed from surrounding gel and stored in 1× PBS at room temperature until sample preparation.

### Sample preparation

Whole brains were embedded in 2 w/v % agarose gels and mounted vertically onto the vibratome stage using super glue. Sample hydration was maintained by submerging agarose block in 1× PBS at room temperature. Coronal slices within the hippocampal region of mouse brains were prepared using a VT1200 S vibratome (Leica Biosystems, Nussloch, Germany) with a blade speed of 0.40 mm s^−1^ and amplitude of 1.00 mm.

### Polymer-tissue optical clearing

Brain slices were placed in clearing buffer, a solution of 200 mM sodium borate buffer and 4 w/v % sodium dodecyl sulfate (SDS) at pH 8.5 and 37 °C, to achieve tissue transparency via passive diffusion. Samples were incubated 10–12 days for 200 μm slices, 18–20 days for 1 mm slices, and 26–28 days for 2 mm slices, with solution changed every 3–4 days. After tissue was clear, samples were stored in 1X PBS at room temperature and fresh solution was changed regularly. Reagents such as Triton-X and FocusClear were not added because it was desirable to calculate the materials properties of the samples without any additional effects other than the polymerization product itself, and samples were not used for thick-section optical imaging requiring refractive index homogenization.

### In vitro hydrogel synthesis

To generate comparative samples that lacked brain tissue, hydrogel precursor solutions were polymerized outside of the brain following the CLARITY hybridization protocol described previously. Polymerized gels were handled in the same fashion as hybridized tissue samples.

### Raman spectroscopy

Raman experiments were performed with NTEGRA Spectra Raman spectrophotometer instrument, equipped with a peltier plate-cooled CCD camera in backscattering geometry with 600 mm^−1^ grating. The illumination source was a 473 nm Cobolt BluesTM continuous wave diode-pumped solid state laser brought to the brain samples by a Mitutoyo long working distance objective (100×, 0.7 NA) or to in situ gels by Nikon Plan Fluor (10×, 0.3 NA). Laser power at sample was 2 mW, as measured by Coherent LaserCheckTM. For all brain scans, samples were measured in a hydrated state, where the sample was sealed under a coverslip in a solution of 1× PBS. Single point scans were acquired with a laser exposure time of 60 s. A minimum of two scans per location were averaged. Raw data was corrected by subtracting the water signal and fluorescence background, and normalized to the peak with the highest intensity, representing CH_2_ rocking at ~ 3000 cm^−1^.

### Correlative Raman and fluorescence 2D spectral maps

Fluorescence and Raman spectral maps were created sequentially using the same Raman spectrometer and the same spectral window, allowing correlation of cellular features with Raman polymer signature. The effective acquisition range for fluorescence was 477–560 nm. To confirm the location of specific cellular components within the polymer-tissue composite, 200-um thick samples were treated overnight with rabbit polyclonal antibodies to parvalbumin at a 1:100 dilution (Abcam, USA), followed by another overnight treatment of AlexaFluor 488 AffiniPure Goat Anti-Rabbit IgG (H + L) secondary antibodies at a 1:100 dilution (Jackson ImmunoResearch). Fluorescence maps of 100 × 100 μm areas were collected with 0.1 s exposure time and 1 μm step size to locate the positions of cell bodies. Following that, for Raman spectral maps, a 30 × 30 μm area was scanned with step size of 1 μm and exposure time of 60 s both to maximize signal-to-noise ratio and to minimize fluorescence background by concurrent laser bleaching. The absence of sample degradation was confirmed by absence of differences amongst the individual 1-min spectra over time, besides changes in fluorescence baseline signal. Multiple Raman spectral intensities can be used to predict unknown analyte concentrations of the samples by assuming the intensities are linear combinations of known compound concentrations. Using an in-house fitting software based on multilinear regression analysis (MLR) (Multilinear regression)^33^, and utilizing the Levenberg–Marquardt algorithm, the full Raman spectrums for 2%, 4%, and 12% AAm concentrations were fit to curves of known compounds of 4% polyacrylamide gel, 4% PFA-fixed brain tissue, and water. The fitting process subtracts a polynomial line from raw data to account for background fluorescence, and then calculates the percentage/ratio (coefficients) of pure compound spectra at each data point along curve that is necessary to best fit the experimental data. These coefficients visualized in 2D heatmap plots, which on x–y plane represents map of area scanned, corresponding to a selected area within the fluorescence image, and color represent intensity/amount of specific compound required. To evaluate and compare the polymer concentration outside and inside cell bodies, 10 coordinates outside and 10 coordinates inside of regions correlated with Parvalbumin fluorescence signal were randomly selected. The spectra from each of those coordinates were analyzed to determine the number of counts at the ~ 1110 cm^−1^ polyacrylamide backbone peak (C–C skeletal vibrations). There were 3 cells for each condition tested.

### Rheology measurements

Viscoelastic properties were measured using a DHR-2 rheometer (TA Instruments, New Castle, DE) with a custom-machined 3 mm parallel plate. To ensure a consistent gap and sample overlap between the rheometer plate and stage, a circular 6 mm-diameter tissue punch (TedPella) was used to excise the hippocampus area from 2 mm thick brain slices, forming symmetrical sample ‘disks’ from the same brain region across all tested samples. The number of samples tested per condition was six, across three distinct animals. To obtain G′ and G″, the plate was lowered upon the center of the sample to form a 0.80 mm gap. A constant oscillation strain of 10%, optimized from several strain amplitude sweeps showing 10% strain consistently fell within the linear elastic regime for all AAm concentrations, was applied during a logarithmic frequency sweep from 100 to 0.10 Hz at 5 points per decade and room temperature. G’ was calculated using the average of the values within the linear regime (5 data points from f: 0.1 to 0.63 Hz) of the G’ vs frequency plots. Frequency sweeps were performed at a range of temperatures from 4 to 84 °C with a 420 s equilibration time between temperature changes. During the measurements, the sample was kept always hydrated in 1X PBS. Precursor viscosity measurement were performed on ARES-G2 rheometer with Peltier Solvent Trap and Evaporation Blocker at constant 37 °C, 0.047 mm gap between 40 mm parallel plates at 100 Hz unidirectional shear rate for 15 min (TA Instruments).

### Protein retention assay

Uncleared 500 μm-thick slices were cleared over a total period of 14 days in 1 ml of clearing buffer. 14 days, the concentration of protein washed out from the sample was obtained from the clearing buffer using the Pierce bicinchoninic acid BCA Protein Assay Kit (Pierce Technology, Rockford, IL), and microplate reader. The clearing buffer was replaced after every measurement. There were 8 samples from 4 distinct animals tested for each condition. The total percentage of protein retained was calculated by assuming that the total protein in mouse brain is approximately 8% of mouse total body weight (brain =  ~ 32,000 ug for a 400 mg mouse).

### Permeability assay

Diffusion coefficients of the samples were obtained by measuring the fluorescence intensity of a solution of fluorescently tagged F(ab)′2 secondary antibodies within the sample over time, using a Leica SP5 optical fluorescence microscope. To limit diffusion to a one-dimensional profile, a 250 μm-thick sample with a flat edge was mounted in between a glass slide and coverslip surrounded on three sides by a 200 μm spacer (iSpacers, Sunjin Labs, Taiwan), following established protocol^31^. Only the single flat edge of the sample was exposed to the solution containing the fluorescent probes through the chamber opening. To construct the chamber, spacers were placed adhesive-side down on a 35 mm petri dish. The sample was placed within the spacers, with flat edge parallel and facing the open side. 50 μl of 1× PBS was added to the slice to maintain hydration. A 22 × 22 mm glass coverslip was placed evenly over the top of the slice and spacer, and sealed with KwikSil sealant (World Precision Instruments) on all three closed edges. 100 μl of 0.3 mM solution in 1×  PBS of 100 kDa F(ab)′2 fragment conjugated to AlexaFluor 488 (lab) was pipetted between the coverslip and petri dish to fill the chamber. Bright-field channel was used to properly focus onto the most uniform edge of the sample, with the flat edge of the sample as vertical as possible. Images were recorded at 488 nm wavelength, 30% laser intensity, 5×  objective every 10 min for one hour. The settings for image acquisition were: 1024 × 1024 resolution, line average = 2, frame average = 4, Hz = 200. For each condition, there were 4 samples from 2 distinct animals. ImageJ was used to obtain raw passive diffusion data by vertical summation of fluorescence intensity as a function of distance from the sample edge within a rectangular ROI. First, the set of images was compiled into a stack in order of progressing time. A rectangle containing the edge and surrounding fluid and extending ~ 2500 μm into the sample was drawn manually and applied to all images in the stack. The x-coordinate of the edge of the sample was determined as the position of the maximum change in intensity at the first time point and set as x = 0. The corresponding values for intensity were normalized on a 0–1 scale using Origin Software. Normalized intensity curves as a function of distance up to 2500 μm from the edge and beginning 20 min after the solution was initially injected into the chamber were fit to a generalized solution of Fick’s Law of one-dimensional diffusion^34,35^ using the Matlab curve- fitting function, which produced values for D, the diffusion coefficient, in units of μm^2^ s^−1^.

### Equilibrium swelling assay

The retained hydrogel mass and water content of the hydrogels were evaluated by weighing cleared 500 μm-thick slices (1) when fully hydrated in 1×  PBS and (2) after desiccation in a vacuum overnight. There were 6 samples from 2 distinct animals tested for each condition. An estimate of hydrogel mass retained in brain samples was calculated by normalizing the dry weight of CLARITY samples to the dry weight of PFA-fixed, non-CLARITY samples. The total mass of equilibrium water content was calculated from the mass difference between the swollen and dry weights.

### Scanning electron microscopy

250 μm coronal sections of CLARITY hybrids were fixed overnight in 4% PFA with 2% Glutaraldehyde in 0.1 M sodium cacodylate Buffer (pH 7.3). Samples were treated consecutively with ferrocyanide-reduced OsO4 (1% OsO4 with 1.5% tetrapotassium ferricyanide) (1 h), freshly prepared and syringe-filtered, 1% Thiocarbohydrazied (TCH) (40 min), 2% OsO4 (1 h) and 1% Uranyl Acetate (overnight), before dehydration in a graded ethanol series (50, 70, 90, 100%, 10 min each), followed by 2 × in 100% Acetonitrile (10 min each). Repeated washing with H_2_O (3 × 5 min) was included between steps of staining and before dehydration. Tissue was then infiltrated with 25%, 50% and 75% EMBed (Electron Microscopy Sciences, Hatfield, PA) in Acetonitrile, followed by 100% EMBed (2 × 3 h) and finally embedding in pure EMBed with polymerization for 48 h at 60 oC. Serial ultrathin sections (200 nm each) were collected on conductive (carbon-coated) glass and Si wafer substrates, and visualized with a Zeiss Sigma FESEM (Zeiss Microscopy, Thornwood, NY) operated at 5–7 kV using BSE detection.

### Statistical analysis

The number of samples (n) used in each experiment is noted in the text. Statistical significance has been measured as two-tailed unequal variance t-test: * p < 0.05.

### Animal care

Animal care, husbandry, and euthanasia procedures were conducted in accordance with established institutional and National Institutes of Health guidelines. The animal protocols were approved by the Stanford University Institutional Animal Care and Use Committee.

## Supplementary Information

Supplementary Information.

## Data Availability

Supplementary material for this paper is available online. The datasets generated during and/or analysed during the current study are available from the corresponding author on reasonable request.

## References

[1] Chung K (2013). Structural and molecular interrogation of intact biological systems. Nature.

[2] Tomer R (2014). Advanced CLARITY for rapid and high-resolution imaging of intact tissues. Nat. Protoc..

[3] Hama H (2011). Scale: A chemical approach for fluorescence imaging and reconstruction of transparent mouse brain. Nat. Neurosci..

[4] Ke MT, Fugimoto S, Imai T (2013). SeeDB: A simple and morphology-preserving optical clearing agent for neuronal circuit reconstruction. Nat. Neurosci..

[5] Susaki EA (2014). Whole-brain imaging with single-cell resolution using chemical cocktails and computational analysis. Cell.

[6] Chung K (2013). CLARITY for mapping the nervous system. Nat. Methods.

[7] Richardson D, Lichtman J (2015). Clarifying tissue clearing. Cell.

[8] Poguzhelskaya E (2014). Simplified method to perform CLARITY imaging. Mol. Neurodegener..

[9] Lee SY (2016). APEX fingerprinting reveals the subcellular localization of proteins of interest. Cell Rep..

[10] Yang B (2014). Single-cell phenotyping within transparent intact tissue through whole-body clearing. Cell.

[11] Chen R (2015). Wireless magnetothermal deep brain stimulation. Science.

[12] Epp J (2015). Optimization of CLARITY for clearing whole-brain and other intact organs. eNeuro.

[13] Kulicke WM, Kniewske R, Klein J (1982). Preparation, characterization, solution properties and rheological behaviour of polyacrylamide. Prog. Polym. Sci..

[14] Kjoniksen AL, Nystrom B (1996). Effects of polymer concentration and crosslinking density on rheology of chemically cross-linked poly(vinyl alcohol) near the gelation threshold. Macromolecules.

[15] Baselga J, Hernandez-Fuentes I, Pierola F, Llorente MA (1987). Elastic properties of highly cross-linked polyacrylamide gels. Macromolecules.

[16] Abidine, Y. *et al*. Wide frequency rheological modeling of crosslinked polyacrylamide gels. HAL, hal-00840468v1 (2013).

[17] Stockmayer W (1944). Theory of molecular size distribution and gel formation in branched polymers II. General cross linking. J. Chem. Phys..

[18] Lai HM (2016). Rationalization and validation of an acrylamide-free procedure in three-dimensional histological imaging. PLoS One.

[19] Fallenstein GT, Hulce VD (1989). Dynamic mechanical properties of human brain tissue. J. Biomech..

[20] Nicolle S (2005). Shear linear behavior of brain tissue over a large frequency range. Biorheology.

[21] Ommaya A (1968). Mechanical properties of tissues of the nervous system. J. Biomech..

[22] Bilson L (2001). Large strain behavior of brain tissue in shear: Some experimental data and differential constitutive model. Biotechnology.

[23] Bansil R, Gupta M (1980). Effect of varying crosslinking density on polyacrylamide gels. Ferroelectrics.

[24] Murugan R, Mohan S, Bigotto A (1998). FTIR and polarised Raman spectra of acrylamide and polyacrylamide. J. Kor. Phys. Soc..

[25] Thomas E, Haaland D (1990). Comparison of multivariate calibration methods for quantitative spectral analysis. Anal. Chem..

[26] Giz A, Catalgil-Giz A, Alb A, Brousseau J, Reed WF (2001). Kinetics and mechanisms of acrylamide polymerization from absolute, online monitoring of polymerization reaction. Macromolecules.

[27] Gupta M, Bansil R (1981). Laser Raman spectroscopy of polyacrylamide. J. Polym. Sci..

[28] Lee H, Park JH, Seo I, Park SH, Kim S (2014). Improved application of the electrophoretic tissue clearing technology, CLARITY, to intact solid organs including brain, pancreas, liver, kidney, lung, and intestine. BMC Dev. Biol..

[29] Grattoni C (2001). Rheology and permeability of crosslinked polyacrylamide gel. J. Colloid Int. Sci..

[30] Marx V (2018). Optimizing probes to image cleared tissue. Nat. Methods.

[31] Li J, Czajkowsky DM, Li X, Shao Z (2015). Fast immunolabeling by electrophoretically driven infiltration for intact tissue imaging. Sci. Rep..

[32] Krolewski DM, Kumar V, Martin B (2018). Quantitative validation of immunofluorescence and lectin staining using reduced CLARITY acrylamide formulations. Brain Struct. Funct..

[33] Malkovskiy A (2021). The Effect of ethanol consumption on composition and morphology of femur cortical bone in wild-type and ALDH2*2-homozygous mice. Calcified Tissue Int..

[34] Pavesi L, Rigamonti A (1995). Diffusion constants in polyacrylamide gels. Phys. Rev. E..

[35] Sykova E, Nicholson C (2008). Diffusion in brain extracellular space. Physiol. Rev..

