# Supplementary material for: Visualization of the distribution of covalently cross-linked hydrogels in CLARITY brain-polymer hybrids for different monomer concentrations
Source: Sci Rep. 2022 Aug 8;12:13549. doi: 10.1038/s41598-022-17687-x (PMC9360022; doi:10.1038/s41598-022-17687-x)
Supplement: Supplementary file 1 — Supplementary Information. [file 41598_2022_17687_MOESM1_ESM.pdf]

Supplemental Information for:

Visualization of the distribution of covalently cross-linked hydrogels in CLARITY brain-polymer hybrids for different monomer concentrations.

Andrey V. Malkovskiy,‡*[a] Ariane Tom,‡[b] Lydia-Marie Joubert[c] and Zhenan Bao*[b]

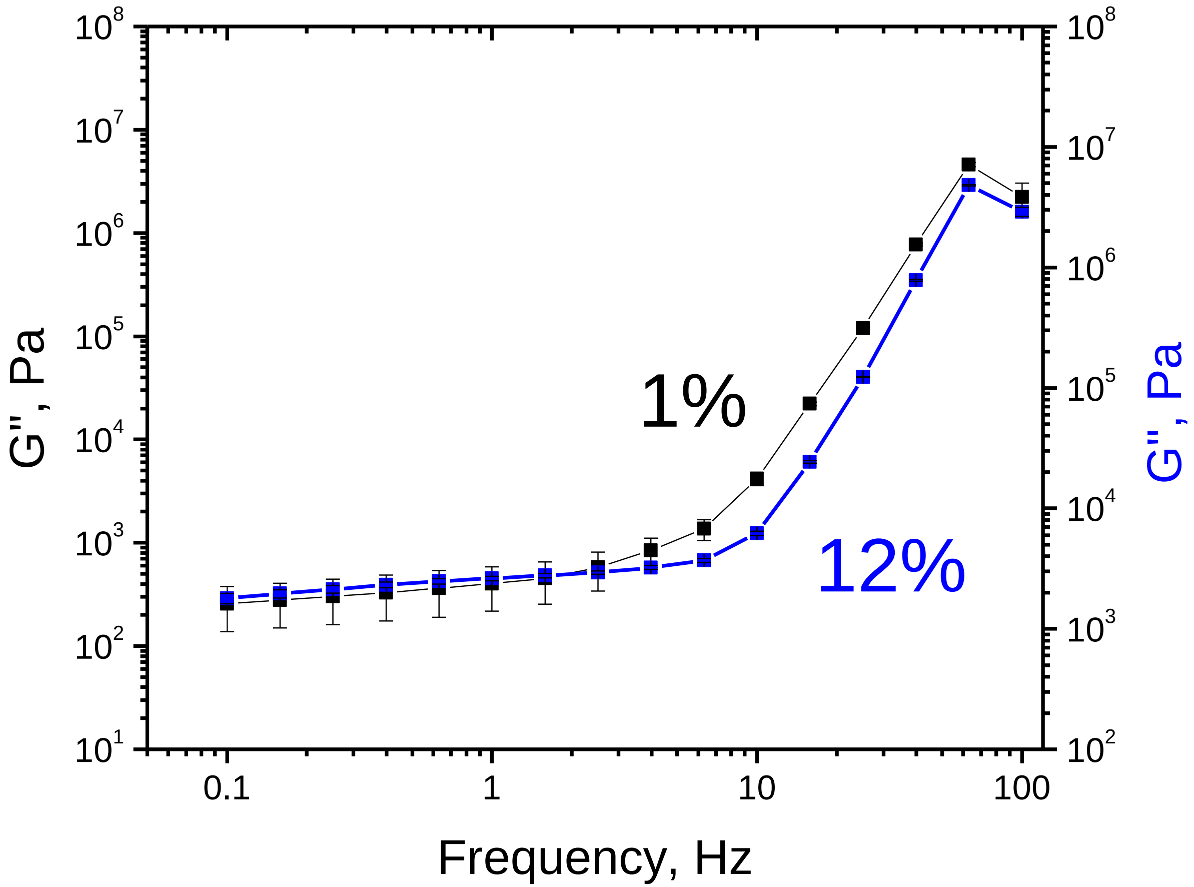

Supplemental Figure 1. Comparison of loss moduli for 1% and 12% AAmPA gels (n=3), showing the shift in onset of stiffening at higher frequencies. This implies a somewhat lower degree of crosslinking for the 12% gel.

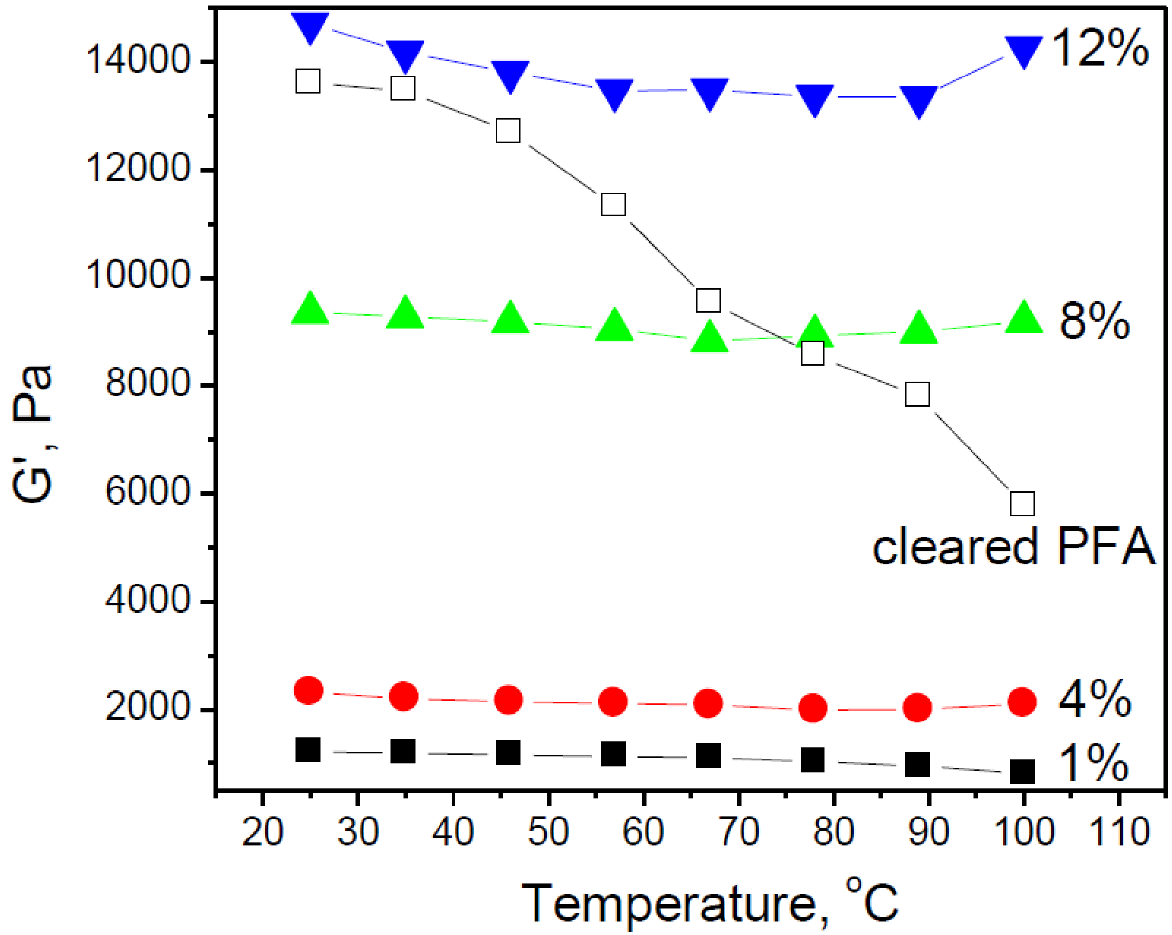

Supplemental Figure 2. Storage moduli at 0.1 Hz for 1%, 4%, 8%, 12% AAmCLARITY mouse brains and cleared PFA-only fixed brain.

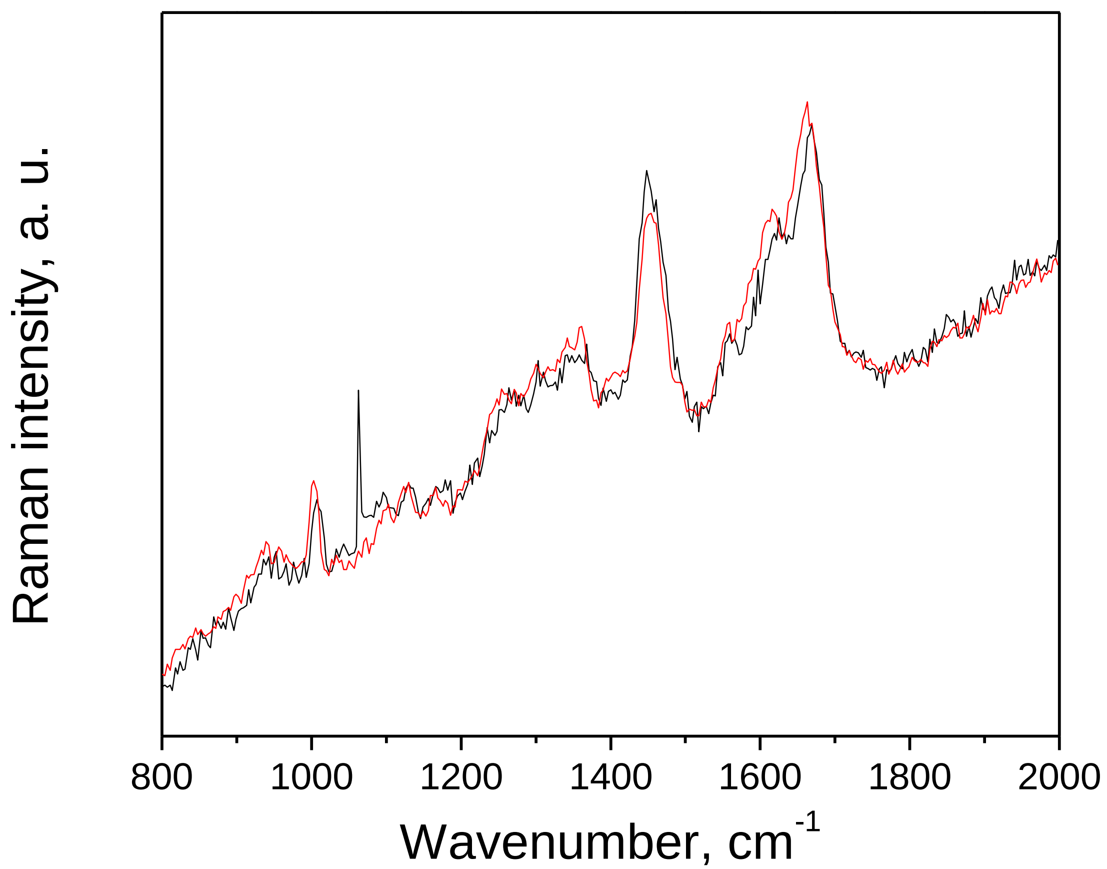

Supplemental Figure 3. Examples of MLR curve fitting results for the spectral range with most prominent features: black line is raw Raman spectral data, red line is the result of MLR fitting algorithm, i.e. a sum of polymer-rich and polymer-poor brain control spectra. Spike in the black line is camera noise, however it does not noticeably affect the quality of the MLR fit, as it showed up only for a few pixels on X scale.

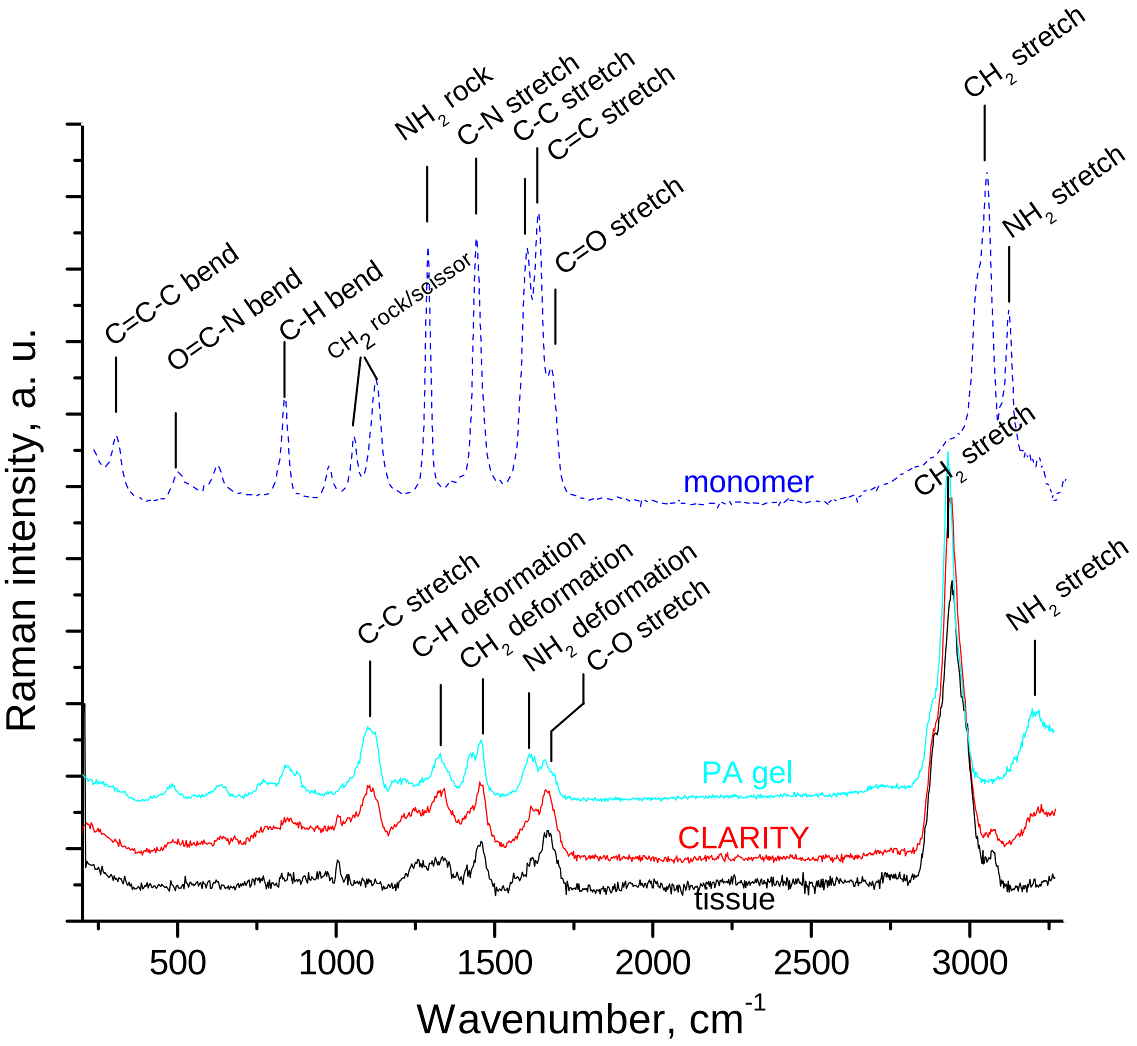

Supplemental Figure 4. Assignment of bond vibrations in Raman spectra of acrylamide monomer, PA gel, 4% AAmCLARITY cleared brain tissue and non-CLARITY cleared brain tissue. Background has been corrected for water and fluorescence.

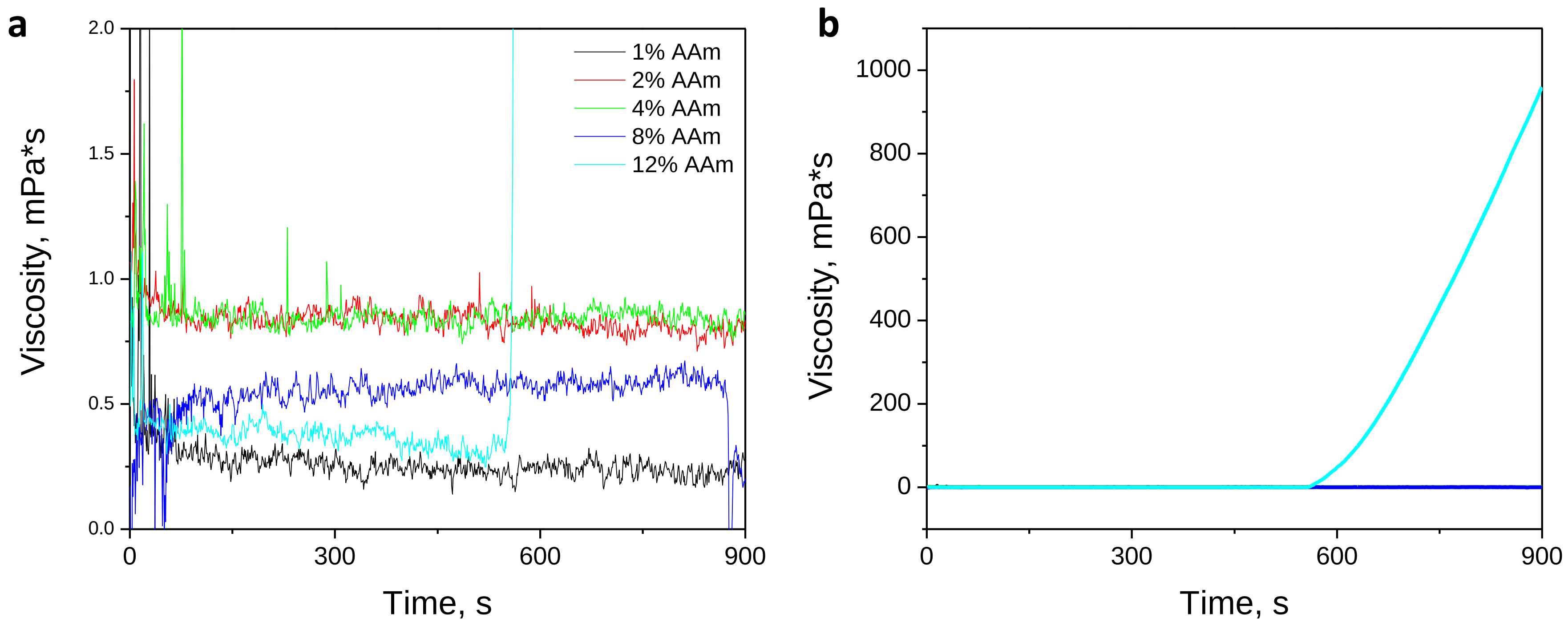

Supplemental Figure 5. Change in solution viscosity at 37 °C at 100 Hz shear rate (**a**), a zoomed-out view is presented in (**b**) to highlight the fast increase in viscosity for 12% gel precursor sample.
